# ROCK1 is a novel Rac1 effector to regulate tubular endocytic membrane formation during clathrin-independent endocytosis

**DOI:** 10.1038/s41598-017-07130-x

**Published:** 2017-07-31

**Authors:** David Soriano-Castell, Albert Chavero, Carles Rentero, Marta Bosch, Maite Vidal-Quadras, Albert Pol, Carlos Enrich, Francesc Tebar

**Affiliations:** 1 Departament de Biomedicina, Unitat de Biologia Cel·lular, Centre de Recerca Biomèdica CELLEX, Institut d’Investigacions Biomèdiques August Pi i Sunyer (IDIBAPS), Facultat de Medicina, Universitat de Barcelona, Casanova 143, 08036 Barcelona, Spain; 2 Institució Catalana de Recerca i Estudis Avançats (ICREA), 08010 Barcelona, Spain

## Abstract

Clathrin-dependent and -independent pathways contribute for β1-integrin endocytosis. This study defines a tubular membrane clathrin-independent endocytic network, induced with the calmodulin inhibitor W13, for β1-integrin internalization. This pathway is dependent on increased phosphatidylinositol 4,5-bisphosphate (PI(4,5)P_2_) levels and dynamin activity at the plasma membrane. Exogenous addition of PI(4,5)P_2_ or phosphatidylinositol-4-phosphate 5-kinase (PIP5K) expression mimicked W13-generated-tubules which are inhibited by active Rac1. Therefore, the molecular mechanisms downstream of Rac1, that controls this plasma membrane tubulation, were analyzed biochemically and by the expression of different Rac1 mutants. The results indicate that phospholipase C and ROCK1 are the main Rac1 effectors that impair plasma membrane invagination and tubule formation, essentially by decreasing PI(4,5)P_2_ levels and promoting cortical actomyosin assembly respectively. Interestingly, among the plethora of proteins that participate in membrane remodeling, this study revealed that ROCK1, the well-known downstream RhoA effector, has an important role in Rac1 regulation of actomyosin at the cell cortex. This study provides new insights into Rac1 functioning on plasma membrane dynamics combining phosphatidylinositides and cytoskeleton regulation.

## Introduction

Endocytosis is an essential process for eukaryotic cells to internalize growth factors, hormones, and nutrients from the plasma membrane (PM) or extracellular fluid^1–4^. The internalization routes can be classified into clathrin-dependent endocytosis (CDE) and clathrin-independent endocytosis pathways (CIE)^2, 5–9^. CIE pathways include different ways of internalization which show high complexity, though all generally share their association with PM microdomains enriched in cholesterol and glycosphingolipids^10–13^. The molecular machinery that regulates these different routes is only now beginning to emerge. In comparison with CDE pathways, the morphological features of membrane carriers generated by CIE pathways range from small vesicles to membrane tubular networks of different size and extension^9, 11, 14–16^.

Several laboratories, including ours, have recently described the existence of PM tubular networks belonging to CIE pathways, with tubules of tens of micrometers in length^11, 15, 17^. Major histocompatibility complex I (MHCI) has been reported to be internalized via Arf6-dependent, clathrin and caveolae-independent endocytosis^18^, and both MHCI and Arf6 were detected in tubules that lack the CDE marker transferrin^15^. Induced non-clathrin-mediated tubular membrane invaginations have also been reported for the uptake of Cholera and Shiga toxins, and the simian SV40 virus^16, 17, 19^. Formation of these tubules seems to require an intact microtubule network^15, 16^. Moreover, we have demonstrated the involvement of Rac1, calmodulin (CaM), and phosphatidylinositol 4, 5-bisphosphate (PI(4,5)P_2_) in this process^15^. While expression of the constitutively active Rac1 mutant Rac1^G12V^ completely abolishes membrane tubules, the dominant negative mutant Rac1^T17N^ triggers the formation. The same phenotype is generated by phosphatidylinositol 4-phosphate-5-kinase (PIP5K) overexpression or by treatment with the CaM inhibitor N-(4-aminobutyl)-5-chloro-2-naphthalenesulfonamide (W13), which increase PI(4,5)P_2_ levels at the PM^15^. Involvement of PI(4,5)P_2_ in the initiation of endocytic events is determined by its ability to bind and recruit several membrane-bending proteins such as dynamin or BAR-domain containing proteins, but also by its role in actin dynamics at the cell surface^20–25^. Afterwards, the decrease of PI(4,5)P_2_ by specific phosphatases and/or phospholipases, such as synaptojanin or phospholipase C (PLC), is important to promote pinch-off of the plasma membrane and the consequent endocytic vesicle production^26–28^.

The small GTPases, Rac1, RhoA, and Cdc42, are implicated in the regulation of several CIE pathways. Rac1 and RhoA control interleukin-2 receptor (IL-2R) uptake^29, 30^, and Rac1 regulates macropinocytosis with Cdc42, which is also required during clathrin-independent carrier (CLIC) and GPI-enriched endocytic compartment (GEEC) endocytosis^31–34^. Several CIE pathways also require Pak1, Pak2, or cortactin activity, which are Rac1 actin-related targets^30, 35^, suggesting that Rac1-dependent actin polymerization plays a key role during these events. The PI(4,5)P_2_-binding protein dynamin, as well as cortactin, have been reported to be important actin-modulating and membrane-remodeling factors during both CDE and CIE^35–37^. Therefore, cortactin and dynamin may be acting downstream of Rac1 to regulate the endocytic tubules formation. Moreover, recent studies have identified myosins regulating endocytosis^38–41^, and it has been shown that an increased assembly of actomyosin networks at the PM antagonizes membrane invagination and endocytosis^42, 43^. Actomyosin is mainly regulated by RhoA through its effector ROCK1 (rho associated coiled-coil containing protein kinase 1), but also by Rac1, and these two GTPases usually have opposite effects in several cellular processes^44^. The possible contribution of Rac1-dependent actomyosin regulation to CIE has not been investigated in depth, and nor has its contribution to tubule regulation. Actually, Rac1 could control tubule outcomes by regulating PI(4,5)P_2_ levels (via PLC activity) and cytoskeleton dynamics (through actin polymerization and myosin activation)^45–51^.

In the present study we demonstrate that increased PI(4,5)P_2_ levels trigger dynamin-dependent endocytic tubules formation and enhance β1-integrin internalization, and that this process can be neutralized by Rac1 activation. We show that Rac1 regulates PM endocytic tubule formation by controlling PI(4,5)P_2_ levels, actin dynamics and myosin activation through activation of PLC, cortactin and ROCK1, respectively. Importantly, the results reveal ROCK1 as a new Rac1 effector and here we propose a novel Rac1-dependent ROCK1 activation pathway to regulate membrane dynamics.

## Results and Discussion

### Integrin internalization via a clathrin-independent, Rac1-regulated endocytic pathway

**Figure 1:**
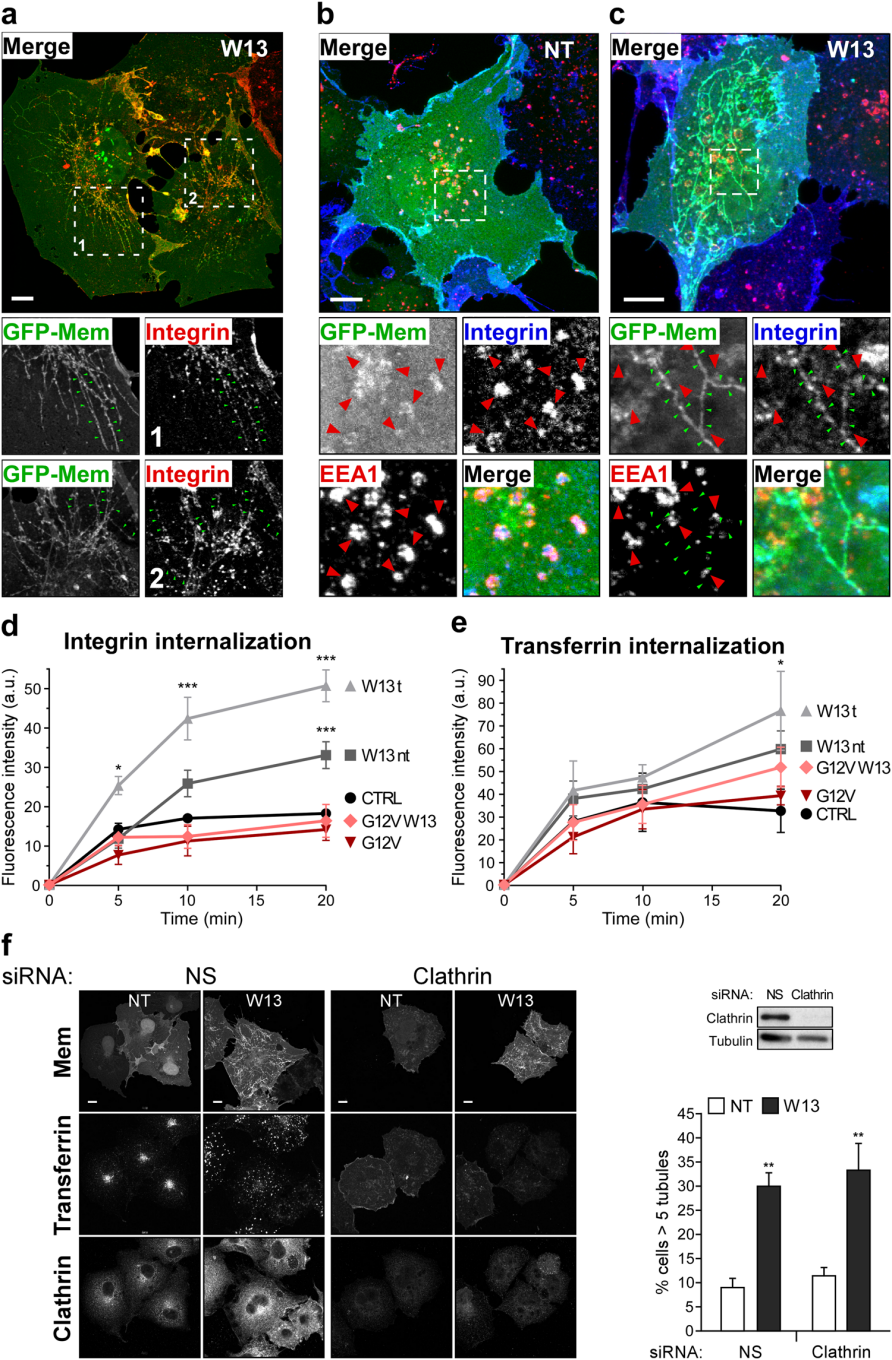
W13-induced PM tubules provide an internalization pathway for β1-integrin. (**a**) COS1 cells grown on coverslips expressing the membrane marker GFP-mem were incubated with an anti-β1-integrin rat antibody for 30 minutes at 4 °C to avoid endocytosis, followed by incubation for 10 minutes at 37 °C to allow internalization in the presence of W13 (20 min, 4.5 µg/ml). After fixation, β1-integrin was detected with an Alexa-555 labeled anti-rat antibody, and images were acquired with a confocal microscope (Leica TCS SP5). The higher magnification insets show β1-integrin localization in W13-induced tubules (green arrowheads). (**b**,**c**) Following the same procedure explained as in (**a**), β1-integrin was detected with an Alexa-647 labeled anti-rat antibody and EEA1 with a specific antibody and the secondary Alexa-555 anti-mouse in untreated (**b**) or W13-treated cells (**c**). Insets show β1-integrin in EEA1-positive endosomes (red arrowheads) (**b**) or in tubules (green arrowheads) (**c**) (bars, 10 µm). (**d**,**e**) Quantification of internalized β1-integrin (**d**) and transferrin (**e**), as explained in the *Materials and Methods*, in COS1 cells expressing GFP-mem or GFP-Rac1^G12V^ for the indicated conditions (W13t, cells presenting tubules; W13nt, cells without tubules). Mean values ± standard error of the mean (SEM) from three independent experiments are shown. Statistical significance between different conditions and the corresponding control was determined by the two-way ANOVA, *p < 0.05, ***p < 0.001. Statistical significance in integrin internalization assay (d) between W13t and W13nt at 5, 10 and 20 minutes were *p < 0.05, ***p < 0.001, ***p < 0.001, respectively. (**f**) COS1 cells co-transfected with GFP-mem and a specific clathrin heavy chain siRNA or a non-specific siRNA as a control (72 h) were incubated with transferrin-TRITC during 15 minutes at 37 °C in the presence or absence of W13 (4.5 µg/ml). After fixation, clathrin was detected with an anti-mouse antibody (clone ×22) and the corresponding Alexa-647 secondary antibody. Confocal images were acquired with a confocal microscope (Leica TCS SP5) through the corresponding channels (bars, 10 µm). Downregulation of clathrin expression by its specific siRNA is shown by western blotting using a rabbit polyclonal antibody Graph shows the percentage of cells presenting tubules (>5 tubules/cell) in the indicated conditions. Mean values ± standard error of the mean (SEM) from three independent experiments are shown. Statistical significance between W13-treatment and the corresponding control was determined by the paired Student’s *t*-test, **p < 0.01.

We have previously shown that Rac1 activity can regulate the formation of membrane tubular structures, with the dominant negative Rac1 increasing and the constitutively active mutant reducing the percentage of cells presenting tubules^15^. These tubular membrane structures, which are also induced after treatment with the calmodulin inhibitor W13, transported clathrin independent endocytic cargoes like MHCI^15^. Since Rac1 activity can control integrin trafficking, and vice versa^52–56^, we have examined whether integrins were also present in these endocytic tubules and if the presence of such tubules affects integrin transport to early endosomes (EEs). COS1 cells were incubated with an antibody that recognizes the β1-integrin ectodomain and treated then with W13 for 10 minutes at 37 °C, before fixing and immunostainning cells with anti-EEA1 antibody. The images in Fig. 1a show the presence of β1-integrin (red) in W13-induced tubules, visualized with the expressed membrane marker GFP-mem (green). Whereas β1-integrin was clearly detected in EEA1-positive endosomes of control cells (Fig. 1b), those cells that contained extensive tubulation (W13 treated) showed low β1-integrin labeling in EEs (Fig. 1c). Considering the presence in tubules as internalized molecules, cells exhibiting tubules showed increase in the total internalized β1-integrin after treatment with W13 at different time points (5, 10 and 20 min) compared with control cells (Fig. 1d). In these settings, the effect of W13-induced tubules on transferrin internalization, a well-established cargo of the CDE route, was also analyzed (Fig. 1e). Transferrin was not observed in W13-tubules and increased transferrin internalization was observed only at 20 minutes in W13-induced tubules compared to control cells, which could be explained by the previously reported effect of W13 inhibiting sorting from early endosomes^57–60^ and consequently transferrin recycling that at later time points contributes to the uptake measurements. Likewise, this could be the reason for increased β1-integrin internalization at later time points in W13-treated cells not presenting tubules. To corroborate that clathrin did not participate in W13-induced tubule formation, clathrin expression was inhibited by siRNA knockdown (Fig. 1f). Figure 1f shows that clathrin downregulation did not modify the extend of W13-induced tubules and, in agreement with the inhibition of recycling, W13 treatment accumulated transferrin in EEs vesicles at the cell periphery in contrast to its perinuclear localization observed in control cells. These results indicate that induced tubular endocytic membrane structures are a cellular port of entry important for β1-integrin internalization in a CIE pathway.

The effect of W13 on β1-integrin internalization was simultaneously analyzed in cells expressing the constitutively active Rac1 mutant (GFP-Rac1^G12V^). Image quantification showed that Rac1^G12V^ expression completely abrogated the W13-increased β1-integrin internalization at all-time points analyzed (Fig. 1d), indicating that active Rac1 blocks tubule formation instead of promoting tubule fission. Besides, the results imply that Rac1 is a β1-integrin internalization regulator, and suggest that it may regulate integrin turnover through CIE.

### Induction of dynamin-dependent tubules in CIE pathway by increased PI(4,5)P_2_

**Figure 2:**
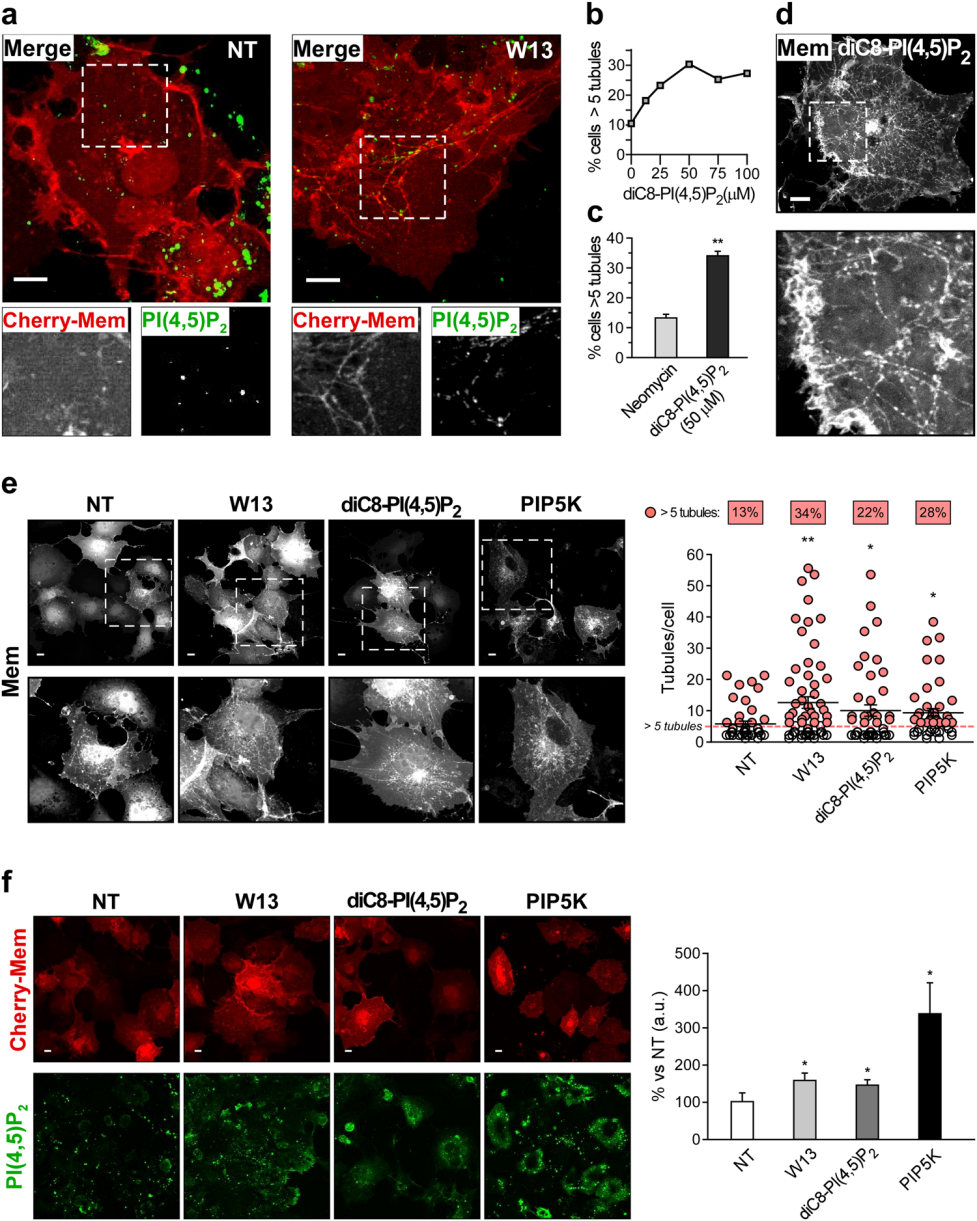
Increased levels of PI(4,5)P_2_ induces membrane tubules at the PM. (**a**) COS1 cells expressing Cherry-Mem grown on coverslips were incubated with W13 (20 min, 4.5 µg/ml). After fixation (PFA 4%, 15 min at 37 °C), endogenous PI(4,5)P_2_ was detected with a specific antibody and the corresponding anti-mouse Alexa-488 labeled secondary antibody. The images were acquired with a confocal microscope (Leica TCS SP5) and magnification insets show the presence of PI(4,5)P_2_ in W13-induced membrane tubules. (**b**) The percentage of cells presenting more than 5 tubules was determined after 20 min of diC8-PI(4,5)P_2_ dose-response at the indicated concentrations. (**c**) The percentage of cells with tubules after 20 min incubation with 50 µM diC8-PI(4,5)P_2_ or neomycin control. (**d**) Confocal image of cherry-mem depicted tubules in cells treated with diC8-PI(4,5)P_2_ as in (**c**) (bars, 10 µm). (**e)** In Cherry-Mem expressing cells, the percentage of cells presenting tubules and the number of tubules per cell were determined in different experimental conditions: W13 (20 min, 4.5 µg/ml), diC8-PI(4,5)P_2_ (20 min, 50 µM) or GFP-PIP5K expression. Statistical significance between the different conditions and the control (NT, non-treatment) was determined by the unpaired Student’s *t*-test, **p < 0.01 (n = 100 cells per condition). Representative confocal images acquired through the red channel and insets displaying cells presenting cherry-Mem decorated tubules are shown (bars, 5 µm). (**f**) In the same experimental conditions as in (**e**), the intracellular levels of PI(4,5)P_2_ were determined by immunofluorescence performed as in (**a**). Graph shows the percentage of fluorescence (a.u., arbitrary units) of each condition vs NT, mean values ± standard error of the mean (SEM) from three independent experiments. Statistical significance between the different conditions and the NT was determined by the paired Student’s *t*-test, *p < 0.05. Representative z-stacks confocal projection images are shown (bars, 10 µm).

Next, clathrin-independent endocytic tubules were further characterized and we focused on the molecular mechanisms activated by Rac1 and its regulation. We and others reported that membrane tubules are induced after increasing PI(4,5)P_2_ levels by overexpression of PIP5K^15, 61^. In fact, W13-induced PM tubules appear to depend on PIP5K activity^15^. The presence of PI(4,5)P_2_ in W13-tubules was confirmed by immunostaining with an anti-PI(4,5)P_2_ antibody (Fig. 2a). Moreover, tubule induction by PI(4,5)P_2_ increase was supported by a dose response curve with exogenous diC8-PI(4,5)P_2_ (previously conjugated with the neomycin carrier for its transmembrane delivery)^62, 63^ (Fig. 2b). Addition of 50 µM of diC8-PI(4,5)P_2_ increased the percentage of cells with tubules up to approximately 35% compared to 12% observed by the neomycin carrier in control cells (Fig. 2c,d). Similar ratio elicited by W13 treatment was observed by overexpression of PIP5K or diC8-PI(4,5)P_2_ incubation (Fig. 2e). In addition, these different experimental conditions similarly increased both the number of tubules per cell (Fig. 2e), and PI(4,5)P_2_ levels detected by immunofluorescence compared to control cells (Fig. 2f). These results demonstrated a direct relationship between increased PI(4,5)P_2_ levels and tubule development. Therefore, W13-treatment could be used to increase PI(4,5)P_2_ levels and tubulation at the PM.

**Figure 3:**
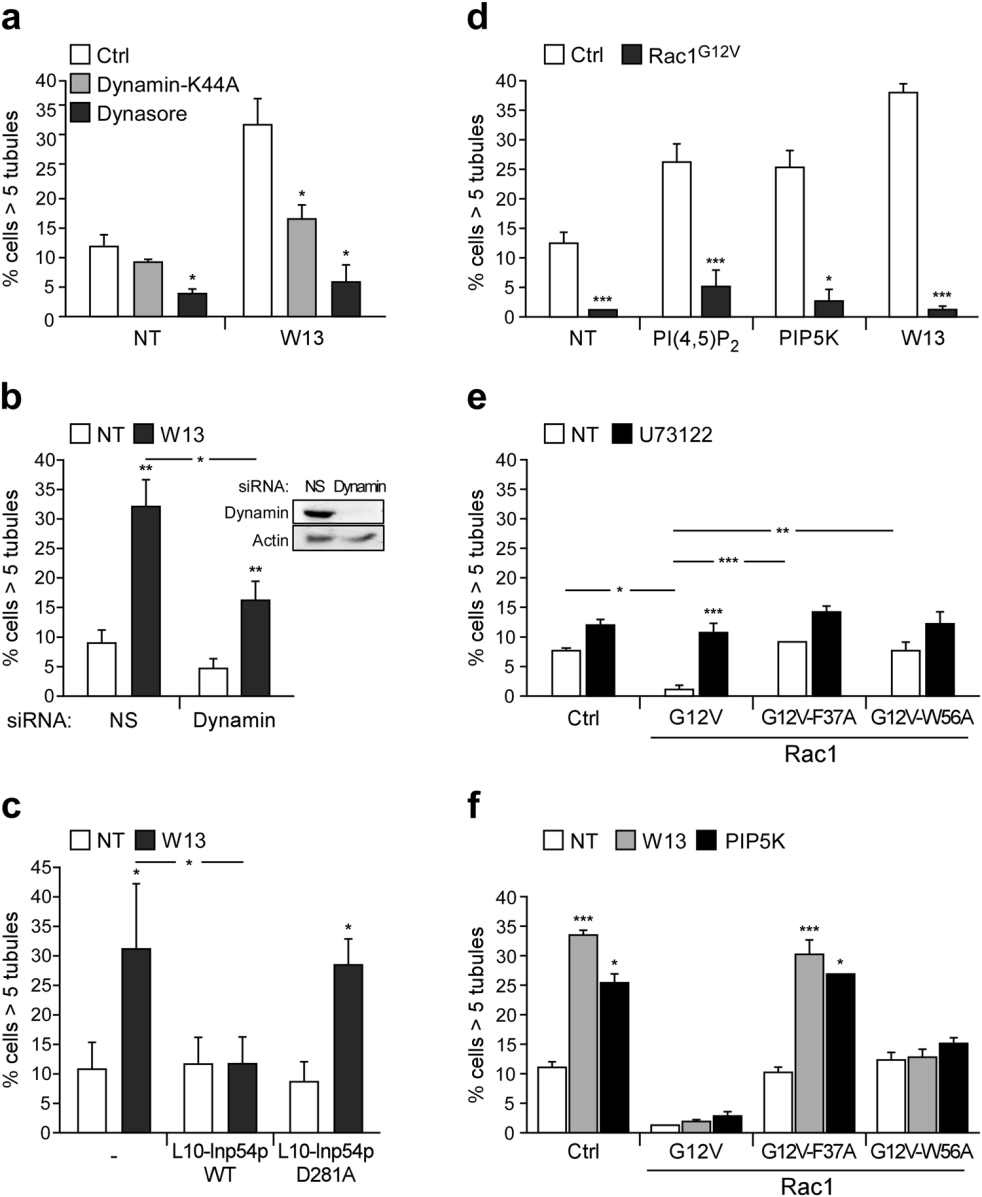
PI(4,5)P_2_-induced tubulation is dynamin-dependent and its inhibition by active Rac1 involves PLC activity. (**a**,**b**) In the presence or absence of W13 (20 min, 4.5 µg/ml), the percentage of cells with tubules was determined after dynasore treatment (30 min, 150 µM) or dynamin^K44A^ expression (24 h) (**a**) and in cells transfected with a specific dynamin siRNA or a non-targeting siRNA as a control (48 h) (**b**). Downregulation of dynamin expression by its specific siRNA is shown by western blotting. (**c**) The percentage of cells with tubules was determined in cells expressing Cherry-Mem alone or co-expressed with L10-GFP Inp54p or L10-GFP Inp54p^D281A^, in the presence or absence of W13 (20 min, 4.5 µg/ml). (**d**) The percentage of cells with tubules was determined in 1-hour starved cells expressing Cherry-Mem or Cherry-Rac1^G12V^ in the presence or absence of PI(4,5)P_2_ (20 min, 50 µM), W13 (20 min, 4.5 µg/ml), or GFP-PIP5K expression. (**e**,**f**) The percentage of cells displaying tubules among the starved cells expressing GFP-mem, GFP-Rac1^G12V^, or active Rac1 mutants (F37A and W56A) after the treatment with the PLC-inhibitor U73122 (20 min, 5 µM) (**e**), W13 (20 min, 4.5 µg/ml), or PIP5K overexpression (**f**). Mean values ± SEM from three independent experiments are shown in all cases. Statistical significance between different conditions and the corresponding controls were determined by Student’s *t*-test, *p < 0.05, **p < 0.01, ***p < 0.001.

It is known that increased PI(4,5)P_2_ are necessary for endocytosis to proceed because they recruit several PI(4,5)P_2_-binding proteins, including adaptor proteins, BAR-domain containing proteins, and dynamin (among others)^20^. Although dynamin has an important role in the scission of endocytic vesicles from the PM, it has also been involved in membrane deformation and tubular membrane organization^24, 64–68^. Therefore, we investigated the role of dynamin in these PI(4,5)P_2_-induced tubules. Dynamin action was inhibited by dominant negative mutant expression (dyn^K44A^; Fig. 3a), treatment with a specific inhibitor (dynasore; Fig. 3a), or by siRNA knockdown (Figs 3b and 2f). In each of these experimental settings, W13-induced tubules were prevented, indicating that this tubular endocytic pathway is dynamin-dependent. Dynamin was necessary to initiate tubule formation, but an additional role of dynamin in the fission of tubules cannot be discarded.

Dynamin participates in membrane invagination in combination with BAR-domain containing proteins^43, 67^. In W13-induced tubules we have also observed the presence of PACSIN2, an F-BAR-domain protein that binds dynamin, PI(4,5)P_2_ and Rac1^69, 70^. Interestingly, although PACSIN2 interacts with dynamin, it does not bind or colocalize with clathrin^71^. Moreover, in agreement with the report by Kreuk *et al*.^69^, we showed that expression of the active Rac1 mutant inhibited the presence of PACSIN2-positive tubules in COS1 cells after W13 treatment (Supplementary Fig. S1). In addition, it has been reported that PACSIN2 regulates caveolae biogenesis and endocytosis in cholesterol rich and plasma membrane ordered domains^69, 72, 73^, where active Rac1 is located^52, 74^. Actually, we have previously shown that cyclodext rin, a PM cholesterol chelator, inhibited W13-tubule formation^15^. Therefore, we analyzed whether the specific PI(4,5)P_2_ increase elicited by W13 treatment was responsible for tubulation in these domains. Tubules were inhibited in cells expressing a PI(4,5)P_2_-phosphatase specifically targeted to PM ordered domains by the 10 N-terminal amino acids of Lck (L10-GFP-Inp54p)^75^. Otherwise, no effect was observed with the phosphatase dead mutant (L10-GFP-Inp54p^D281A^) (Fig. 3c). Together, these results show that increased PI(4,5)P_2_ levels in specific PM domains, where clathrin-independent and dynamin-dependent endocytosis takes place, are probably responsible for tubule formation.

In agreement with the localization of Rac1 in ordered domains^74, 76^, tubular endocytic membranes present in control cells, or elicited by W13 treatment, were inhibited by active Rac1, as well as tubules induced by PIP5K overexpression or by the addition of exogenous diC8-PI(4,5)P_2_ (Fig. 3d). These results, together with the fact that Rac1^G12V^ expressing cells showed reduced PI(4,5)P_2_ immunostaining in W13-treated cells (Supplementary Fig. S2), prompted us to study the role of Rac1 effectors in tubulation.

### The role of PLC-regulated PI(4,5)P_2_ levels on tubule inhibition by active Rac1

Rac1 can modulate PI(4,5)P_2_ levels at the PM by activating PLC^77, 78^, which hydrolyzes PI(4,5)P_2_ generating diacylglycerol (DAG) and inositol trisphosphate (IP_3_)_._ Then, we analyzed PLC involvement by two strategies: (i) inhibition of PLC activity with its inhibitor U73122, and ii) expression of GFP fusion proteins for two previously described constitutively active (GTP-bound), but PLC-deficient, Rac1 mutants (Rac1^G12V-F37A^ and Rac1^G12V-W56A^)^77^.

Quantification of tubule formation in COS1 cells demonstrates that U73122 impaired the tubule inhibition produced by GFP-Rac1^G12V^ expression (Fig. 3e). Moreover, the expression of both Rac1^G12V-F37A^ and Rac1^G12V-W56A^ did not inhibit tubules in untreated cells (Fig. 3d). These results strongly suggest that PLC plays an important role in Rac1-dependent tubule inhibition.

To further analyze PLC activity involvement in the Rac1-dependent inhibition of tubule formation, we assessed the effect of Rac1^G12V-F37A^ and Rac1^G12V-W56A^ expression on PI(4,5)P_2_-induced tubulation, either by W13-treatment or PIP5K-overexpression. As expected, W13 and PIP5K induced a similar percentage of cells presenting tubules in control and Rac1^G12V-F37A^ expressing cells (Fig. 3f), confirming the involvement of PLC activity. However, the expression of the Rac1^G12V-W56A^ mutant was still able to block tubule formation (Fig. 3f).

Together, these results suggest that, although PLC plays a key role in tubule inhibition by active Rac1, additional mechanism contributes to the inhibition, as revealed through the use of the PLC-deficient mutants. Since Rac1^G12V-F37A^ mutant is not able to translocate cortactin to the plasma membrane or interact with ROCK1 (two important factors for cortical actomyosin regulation)^79, 80^, the Rac1^G12V-W56A^ mutant was therefore considered a valuable tool for studying the role of cytoskeleton in the PLC-independent tubular-inhibitory effect of active Rac1.

### Cortactin-dependent actin polymerization inhibits tubular endocytic membrane structures downstream of active Rac1

Active Rac1 is important to control actin polymerization (F-actin) at the PM and F-actin depolymerizing agents are known to generate membrane tubules in many cell types^81, 82^. Thus, active Rac1, by increasing F-actin at the cell cortex, could inhibit PM invagination and consequently tubule formation. It has been reported that Rac1^G12V-F37A^ mutant is defective in cortical actin generation^79, 83, 84^. Therefore, it is plausible that the different tubule inhibition response observed with both active mutants in this study (F37A and W56A) may be related to their different abilities to regulate actin polymerization.

**Figure 4:**
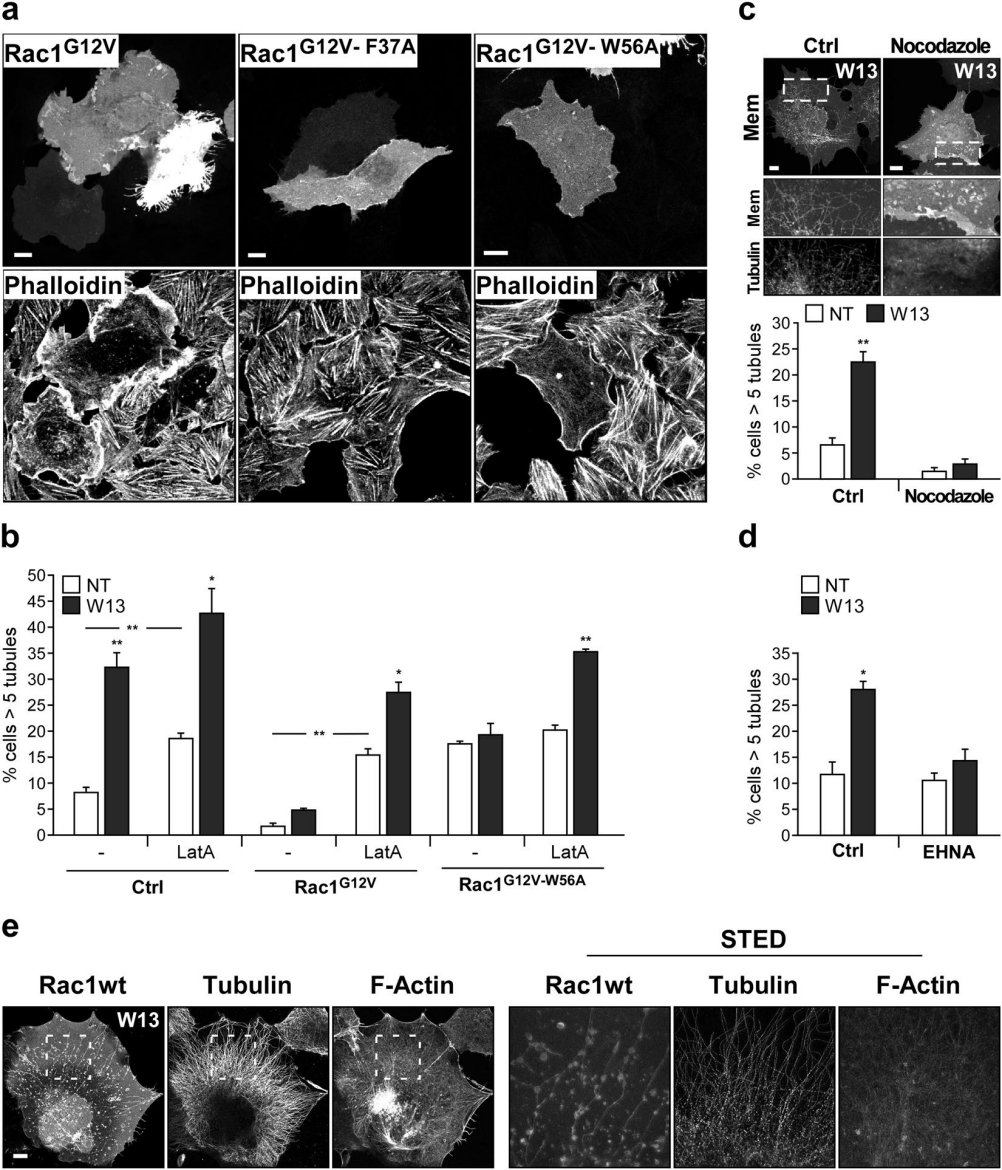
Actin cytoskeleton is involved in Rac1-dependent tubule inhibition. (**a**) Vero cells expressing GFP-Rac1^G12V^, GFP-Rac1^G12V-F37A^, or GFP-Rac1^G12V-W56A^ were grown on coverslips, and F-actin was visualized by confocal microscopy using phalloidin-TRITC (15 min, 0.04 U/ml) (bars, 10 µm). (**b**) The percentage of COS1 cells presenting tubules was determined in starved cells expressing GFP-Rac1^G12V^ or GFP-Rac1^G12V-W56A^ and treated with the F-actin depolymerizing agent latrunculin A (LatA; 20 min, 200 nM) in the presence or absence of W13. (**c**,**d**) Percentage of cells with tubules expressing GFP-mem after pre-incubation with nocodazole (10 min, 30 µM) (**c**) or the dynein inhibitor EHNA (6 hours, 1 mM) (**d**) and incubation with W13 for 20 min. Mean values ± standard error of the mean (SEM) from three independent experiments are shown in all cases. Statistical significance between W13-treatment and the corresponding control were determined by Student’s *t*-test, *p < 0.05, **p < 0.01. (**e**) In cells expressing Venus-Rac1wt and treated with W13, tubulin was detected by immunofluorescence using a mouse anti-β-tubulin antibody and a secondary anti-mouse-Alexa594, and F-actin was detected using SiR actin (SC006). Representative confocal images and STED images from the selected areas are shown (bars, 10 µm).

To determine the effect of these mutants on actin polymerization, F-actin was detected in Vero cells expressing the GFP-Rac1^G12V^, GFP-Rac1^G12V-F37A^, or GFP-Rac1^G12V-W56A^ using conjugated phalloidin-TRITC. Vero cells, which also showed W13-induced PM tubulation^15^, were used instead of COS1 to improve the visualization of actin cytoskeleton. Fluorescence confocal images showed that GFP-Rac1^G12V^ and GFP-Rac1^G12V-W56A^ modified the F-actin pattern by severely reducing stress fibers and increasing cortical F-actin. The effect of G12V was stronger than the G12V-W56A mutant. By contrast, GFP-Rac1^G12V-F37A^ mutant did not affect the actin organization (Fig. 4a).

To establish a possible connection between the increased cortical F-actin and the tubule inhibition produced by the active Rac1 mutants (Rac1^G12V^ and Rac1^G12V-W56A^), actin filaments were disrupted using the depolymerizing agent Latrunculin A (LatA) in W13-treated cells. Actin depolymerization decreased the tubule formation inhibition by Rac1^G12V^ and completely eliminated the inhibitory effect of Rac1^G12V-W56A^ (Fig. 4b). These results indicate that inhibition of membrane tubulation by Rac1 depends on actin polymerization, and that actin cytoskeleton is not necessary for membrane invagination and elongation to proceed. Actually, considering the critical role of microtubules (MTs) for the stabilization of W13-induced tubules described previously^15^, and further analyzed here using the MT depolymerizing agent nocodazole (Fig. 4c), and the recently described role of dyneins for the stabilization and elongation of PM tubular structures^16^, the general dynein inhibitor erythro-9-[3-(2-hydroxynonyl)] adenine (EHNA) impaired W13-induced tubulation in COS1 cells (Fig. 4d). In addition, β-tubulin and F-actin staining in W13-induced tubules cells expressing Venus-Rac1wt, showed some association of these tubules with MTs but not with F-actin (Fig. 4e). Although only occasional coincidence of MTs with tubules was observed, STED and confocal microscopy images revealed highly similar pattern and directionality between both networks (Figs 4e and S4a), which is consistent with the dependency of W13-induced tubules on the integrity of MTs (Fig. 4c). Accordingly, nocodazole also inhibited β1-intergrin internalization elicited by W13 treatment in cells presenting tubules (Supplementary Fig. S4b). These results indicate a key role of dyneins and MTs in PI(4,5)P_2_-induced membrane elongation towards the cell center.

In summary, actin cytoskeleton is unnecessary for tubule elongation (operated by MTs and dyneins) and Rac1-driven actin polymerization is critical to inhibit basal and PI(4,5)P_2_-induced membrane invagination. However, the results obtained with LatA cannot rule out a role of actin polymerization in PM invagination scission.

**Figure 5:**
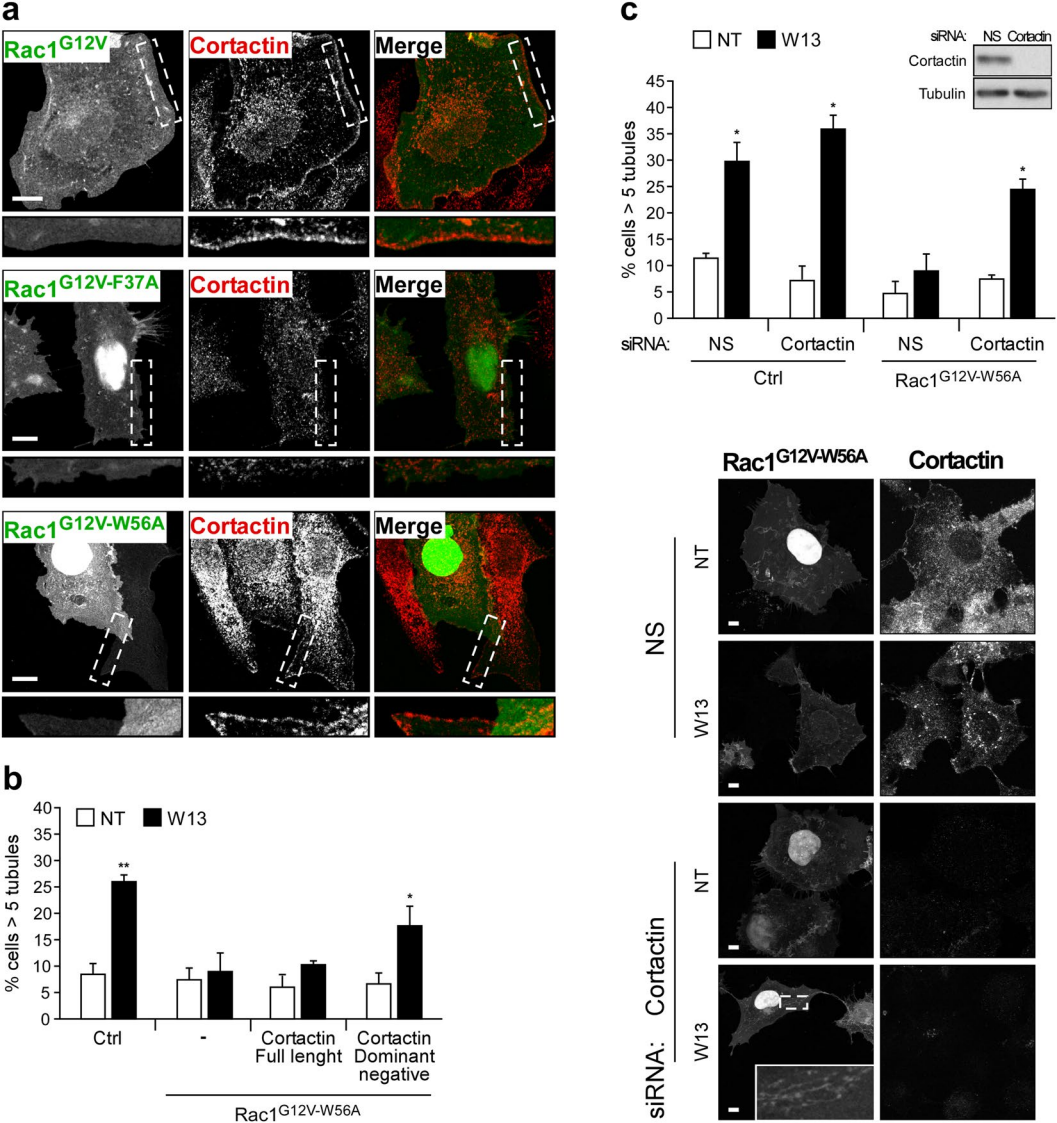
Cortactin is involved in Rac1-dependent tubule inhibition. (**a**) Vero cells grown on coverslips were transfected with GFP-Rac1^G12V^, GFP-Rac1^G12V-F37A^, or GFP-Rac1^G12V-W56A^. After fixation, immunofluorescence was performed to detect endogenous cortactin using an antibody and the corresponding Alexa-555 anti-mouse secondary antibody. Magnification insets show the presence or absence of cortactin at the PM (bars, 10 µm). (**b**) Percentage of COS1 cells presenting tubules, after W13 treatment, in cells transfected with cherry-Rac1^G12V-W56A^ alone or co-transfected with full length cortactin or with a cortactin mutant lacking the PH and SH3 domains, acting as a dominant negative. (**c**) The percentage of cells presenting more than 5 tubules per cell was quantified in untreated (NT) and W13-treated cells co-transfected with GFP-Mem or GFP-Rac1^G12V-W56A^ and the non-specific (NS) or cortactin siRNAs (48 h). Downregulation of cortactin expression by its specific siRNA is shown by western blotting. The W13-induced tubules and cortactin downregulation in GFP-Rac1^G12V-W56A^ and cortactin siRNA-transfected cells is shown by immunofluorescence as in (**a**) (bars, 10 µm). Mean values ± standard error of the mean (SEM) from three independent experiments are shown in all cases. Statistical significance between different conditions was determined by Student’s *t*-test, *p < 0.05, **p < 0.01.

Rac1^G12V-W56A^ and Rac1^G12V-F37A^ have differential effects on cortical F-actin, which may explain the differences in tubule formation inhibition by each mutant. Indeed, it has been described that active Rac1^F37A^ is not able to translocate cortactin, an actin polymerizing protein, to the PM^79^. To address if Rac1^G12V-W56A^ translocates cortactin to the PM to inhibit PI(4,5)P_2_-induced tubule formation, we analyzed the location of endogenous cortactin in Rac1^G12V^, Rac1^G12V-F37A^ and Rac1^G12V-W56A^ expressing Vero cells by immunofluorescence (Fig. 5a). These images showed that Rac1^G12V^ and Rac1^G12V-W56A^ mutants translocate cortactin to the cell periphery (being again more clear in Rac1^G12V^ expressing cells), and Rac1^G12V-F37A^ does not. Additionally, the involvement of cortactin in Rac1^G12V-W56A^-dependent tubule inhibition was examined by overexpression of a dominant negative mutant (cortactin^ΔPHSH3^)^85^. While expression of the wild type cortactin showed no effect, cortactin^ΔPHSH3^ expression restored W13-induced tubules in Rac1^G12V-W56A^ expressing cells (Fig. 5b). The same result was obtained by slencing cortactin expression through siRNA transfection in cells expressing Rac1^G12V-W56A^ and treated with W13 (Fig. 5c), demonstrating that Rac1 needs a functional cortactin to prevent PI(4,5)P_2_-induced tubulation.

### ROCK1 activity inhibits endocytic tubule formation downstream Rac1

**Figure 6:**
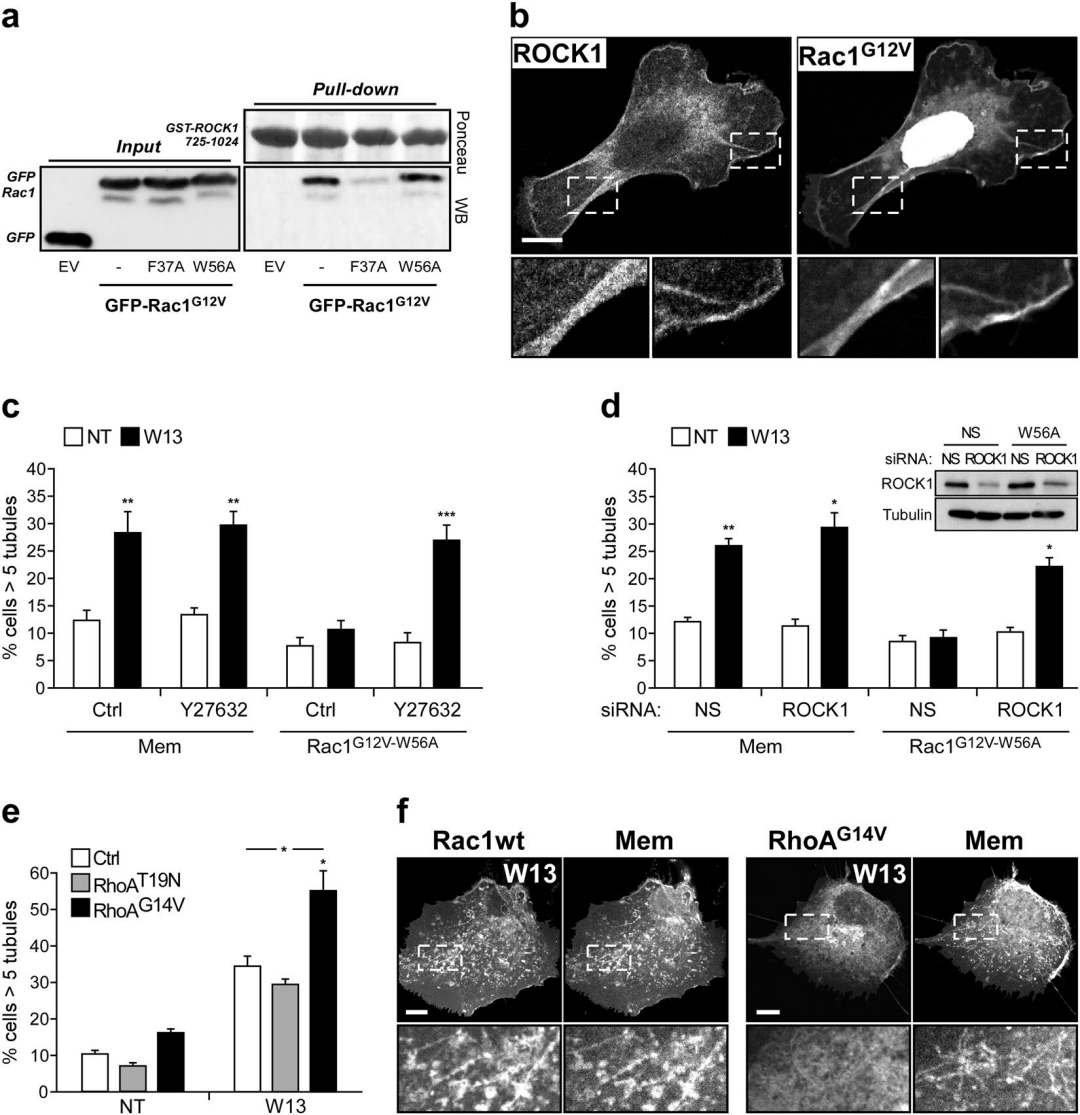
Rac1/ROCK1 pathway prevents tubulation without altering PM localization of cortactin. (**a**) Lysates of COS1 cells expressing GFP-Rac1^G12V^, GFP-Rac1^G12V-F37A^, GFP-Rac1^G12V-W56A^ or the empty GFP-C1 vector (EV) were incubated with immobilized GST-ROCK1^725–1024^ in glutathione Sepharose beads, as described in the *Materials and Methods*. GFP-Fusion proteins present in the input lysates and in the bound fraction were detected by western blotting, using an anti-GFP antibody. A representative western blotting is shown (n = 3). (**b**) Vero cells were co-transfected with cherry-Rac1^G12V^ and GFP-ROCK1. Confocal image insets show regions with high co-localization. (**c**,**d**) COS1 cells were transfected with GFP-Rac1^G12V-W56A^ and treated with or without W13 (20 min, 4.5 µg/ml). The percentage of cells presenting tubules was determined after the inhibition of ROCK1 activity with Y27632 (**c**) or after the inhibition of ROCK1 expression by transfection with a specific siRNA (**d**). Downregulation of ROCK1 expression by its specific siRNA is shown by western blotting. (**e**) The percentage of cells presenting tubules was determined in untreated and W13-treated COS1 cells expressing GFP-RhoA^T19N^, GFP-RhoA^G14V^, or GFP-mem as a control. Mean values ± standard error of the mean (SEM) from three independent experiments are shown in all cases. Statistical significance between different conditions was determined by Student’s *t*-test, *p < 0.05, **p < 0.01, ***p < 0.001. (**f**) Vero cells co-transfected with Cherry-mem and GFP-Rac1 (left panel) or GFP-RhoA^G14V^ (right panel) were treated with W13. Images and insets show localization of GFP-Rac1, but not of GFP-RhoA^G14V^, in the cherry-mem tubules. All images were acquired using the Leica TCS SP5 confocal microscope (bars, 10 µm).

*In vitro* yeast two-hybrid experiments demonstrated that active Rac1 interacted with ROCK1, whereas the active Rac1^F37A^ mutant was defective in such interaction^80^, though the functionality of this association has not been reported yet. ROCK1 is a Ser/Thr kinase that is activated after RhoA-GTP binding to its Rho-binding domain (RBD) due to the release of its autoinhibitory conformation^86^. To analyze the interaction between ROCK1 and the different Rac1^G12V^ mutants, we performed a pull-down assay incubating lysates from GFP-Rac1^G12V^, GFP-Rac1^G12V-F37A^, or GFP-Rac1^G12V-W56A^ expressing cells with GST-ROCK1^725–1024^ immobilized on Sepharose beads. This ROCK1 fragment contains the RBD and a N-terminal portion of its kinase domain that facilitates a proper conformation for RBD/Rho-GTP binding^87^. Western blot analysis showed that Rac1^G12V^ and Rac1^G12V-W56A^ were both pulled down by GST-ROCK1^725–1024^, whereas Rac1^G12V-F37A^ did not (Fig. 6a). Co-immunoprecipitation experiments showing interaction between Rac1^G12V-W56A^ and endogenous ROCK1 were performed as well, although, these results were not consistently reproduced probably due to weak and transient Rac1-ROCK1 interaction and have not been included in this report. In addition, expression of Rac1^G12V^ induced the recruitment and co-localization of ROCK1 at the PM (Fig. 6b). After binding to Rac1-GTP, ROCK1 may be activated and become functional in PM domains where Rac1 is present.

Supporting the hypothesis that ROCK1 is an effector involved in Rac1-dependent tubule formation inhibition, the specific ROCK1 inhibitor Y27632 impaired the inhibition of Rac1^G12V-W56A^ (Fig. 6c). To further confirm ROCK1 involvement in tubule inhibition by the active Rac1 mutant, we silenced ROCK1 expression by siRNA in Rac1^G12V-W56A^ expressing COS1 cells. Downregulation of ROCK1 completely restored the tubules induced by W13 (Fig. 6d). Although ROCK1 could inhibit tubulation by recruiting cortactin to the PM, this possibility was ruled out using Y27632 in Rac1^G12V^- and Rac1^G12V-W56A^-transfected cells. Cortactin translocation appears to be independent of ROCK1 activity (Supplementary Fig. S3), in agreement with others authors^79, 84, 88, 89^. These results indicate that, in addition to cortactin translocation and actin polymerization at the PM, active Rac1impairs tubulation via ROCK1 activity.

However, ROCK1 is a well-known RhoA effector^44, 90^, and to date no functional relationship has been described with other GTPases. In order to exclude RhoA as the upstream activator of ROCK1 responsible for the inhibition of PI(4,5)P_2_-induced tubule formation, the outcome of RhoA activity on W13-induced tubules was examined in COS1 cells by expressing the constitutively active (GFP-RhoA^G14V^) and inactive (GFP-RhoA^T19N^) RhoA mutants. The expression of GFP-RhoA^T19N^ did not modify the percentage of cells presenting tubules neither in control nor W13-treated cells (Fig. 6e). In contrast, when we expressed GFP-RhoA^G14V^ (expected to activate ROCK1), an important and significant increase in tubule-presenting cells was observed after W13-treatment, instead of inhibition (Fig. 6e). RhoA and Rac1 are mutual antagonists^44, 91^, and the observed active RhoA-induced tubulation might be a consequence of endogenous Rac1 inhibition. Moreover, while GFP-Rac1 was present in W13-induced tubules, GFP-RhoA^G14V^ was almost absent (Fig. 6f). Then, RhoA-induced ROCK1 activation takes place in different sites, precluding inhibition of tubule formation by RhoA activity. Together, these data suggest that Rac1/ROCK1, and not RhoA/ROCK1, plays a key role in the inhibition of the endocytic tubule formation.

### Rac1/ROCK1-dependent actomyosin assembly inhibits tubulation

Although there is no reported or conclusive role for ROCK1 as an effector of active Rac1, this protein controls actomyosin downstream of active RhoA. It is feasible, therefore, that by controlling myosin activation, Rac1/ROCK1 interplay could stabilize actin polymerization at the specific sites where tubules should be induced. In turn, this may inhibit tubule formation by mechanical hindrance or by membrane tension increase. It is known that phosphorylation of myosin light chain protein (MLC) is critical for the interaction between myosin and F-actin, and hence for actomyosin generation^92^. Accordingly, MLC phosphatase (MLCP) dephosphorylates MLC and impairs actomyosin formation^93^. In fact, phosphorylation of the MLCP regulatory subunit MYPT1 by ROCK1 results in its inhibition^94–96^. Given that ROCK1 activity may promote actomyosin, we hypothesized that actomyosin induced via Rac1/ROCK1^97^, could be responsible for tubule inhibition.

**Figure 7:**
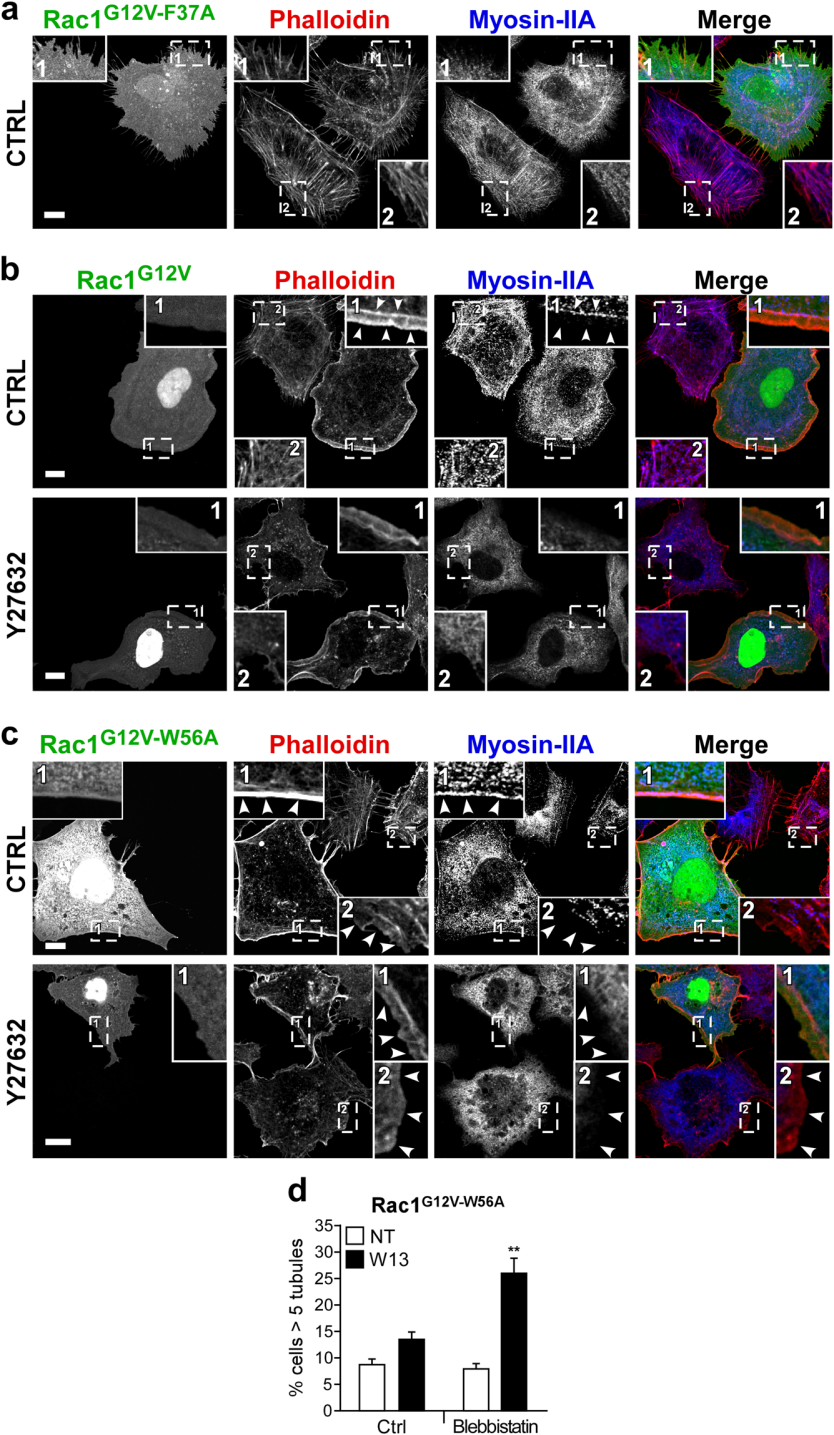
ROCK1 participates in Rac1 induction of actomyosin, and myosin activity is required for Rac1-mediated tubule inhibition. (**a**,**b**,**c**) By immunofluorescence, myosin IIA and F-actin were detected in starved Vero cells expressing GFP-Rac1^G12V-F37A^ (**a**), GFP-Rac1^G12V^ (**b**), or GFP-Rac1^G12V-W56A^ (**c**) after treatment with Y27632 (30 min, 25 µM). The magnification insets show GFP-Rac1, phalloidin-A555, and myosin-IIA-A647 in transfected (1) and non-transfected cells (2). In the cells expressing GFP-Rac1^G12V^ and GFP-Rac1^G12V-W56A^, the images show the loss of stress fibers (and consequently their staining with myosin II) plus myosin recruitment to cortical F-actin (arrow heads), which is reduced after treatment with Y27632 (bars, 5 µm). (**d**) The percentage of cells presenting tubules was determined in the presence or absence of the myosin inhibitor blebbistatin (30 min, 50 µM). Mean values ± standard error of the mean (SEM) from three independent experiments is shown. Statistical significance between different conditions was determined by Student’s *t*-test, *p < 0.05.

To investigate this hypothesis, myosin IIA localization was analyzed by immunofluorescence in untreated or Y27632-treated Vero cells expressing GFP-Rac1^G12V^, GFP-Rac1^G12V-F37A^, or GFP-Rac1^G12V-W56A^. Figure 7 shows that while GFP-Rac1^G12V-F37A^ did not significantly affect myosin IIA localization (Fig. 7a), expression of GFP-Rac1^G12V^ or GFP-Rac1^G12V-W56A^ inhibited myosin IIA stress-fiber localization and enhanced its presence at the cell periphery colocalizing with cortical actin (Fig. 7b,c), being this effect more evident in GFP-Rac1^G12V^ than in GFP-Rac1^G12V-W56A^ expressing cells.

Y27632 treatment inhibited the presence of myosin IIA in both stress fibers and cell periphery in all cells regardless whether they expressed the active Rac1 mutants (Fig. 7), consistent with the restitution of the W13-induced tubules after Y27632 treatment in GFP-Rac1^G12V-W56A^ cells (Fig. 6c). The participation of ROCK1 in myosin IIA localization at the leading edge of wound migrating cells has been previously demonstrated^98^ and the results presented here further support the involvement of ROCK1 in Rac1 induction of actomyosin.

Finally, to clarify the role of myosin activity in tubule inhibition by Rac1, we pre-incubated cells expressing GFP-Rac1^G12V-W56A^ with the general myosin inhibitor blebbistatin before W13-treatment. In this experiment, blebbistatin effectively restored the W13-induced tubules in cells expressing GFP-Rac1^G12V-W56A^ (Fig. 7d). In conclusion, our data establishes ROCK1, for the first time, as a novel downstream effector of Rac1 involved in the control of membrane dynamics via myosin regulation.

### Proposed model of the molecular machinery implicated in the dynamics of PI(4,5)P_2_-induced endocytic tubulation

**Figure 8:**
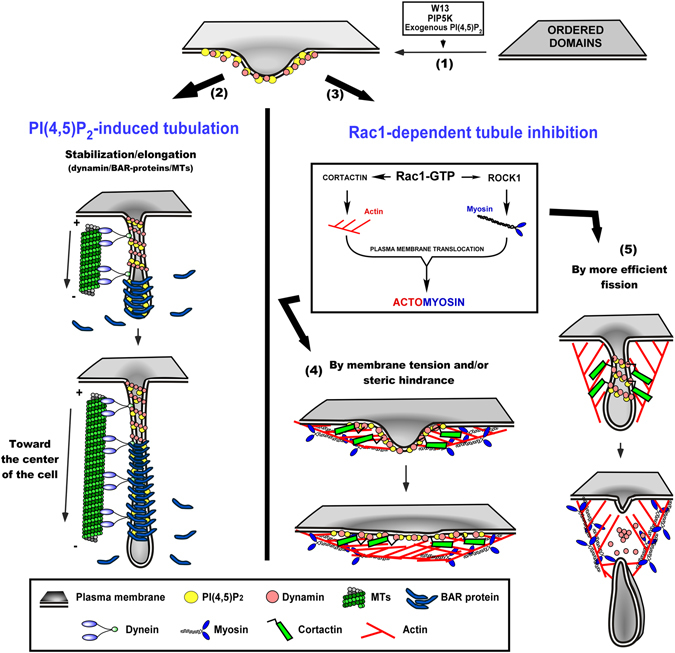
Molecular machinery implicated in the biogenesis and inhibition of PI(4,5)P_2_-induced endocytic tubulation. An increase of PI(4,5)P_2_ in PM ordered domains could induce the recruitment of several PI(4,5)P_2_-binding proteins to generate an incipient membrane deformation (1). When Rac1 activity is low (2), the invagination can be elongated by dyneins toward the center of the cell along microtubules. PI(4,5)P_2_ accumulation, as well as the high degree of membrane curvature, could lead to the recruitment of dynamin or BAR-domain proteins, which in turn, could propagate and stabilize the tubule. By contrast, when Rac1 activity is high, tubulation process could be inhibited by either PLC activation (reducing PIP_2_ levels) or cytoskeleton regulation (inducing cortical actomyosin at the PM) (3). Rac1 appears to stimulate cortactin PM translocation and ROCK1 activity, thereby triggering cortical actin polymerization and association with myosin (i.e., actomyosin). The resulting over-activation of local actomyosin networks could potentially impede tubulation in one of two ways: (i) by forming a local barrier to increasing PM tension or by causing a steric hindrance that impedes the recruitment of tubulating proteins (4); or (ii) by generating mechanical forces needed to pinch off membrane invaginations more efficiently (5).

Taken together, these data support the model summarized in Fig. 8. The regulation of PI(4,5)P_2_ levels in cholesterol rich PM ordered domains is crucial for membrane invagination, elongation and fission, enabling the correct progression of CIE (used for β1-integrin internalization). When PI(4,5)P_2_ levels increase due to CaM inhibition, PIP5K overexpression, exogenous diC8-PI(4,5)P_2_ administration or Rac1 inhibition, a long tubular plasma membrane network is formed (Fig. 8, points 1 and 2). This membrane process requires dynamin, dynein and MTs (point 2). The results presented above demonstrated that activation of Rac1 (overexpression of Rac1^G12V^) inhibits the PI(4,5)P_2_-dependent tubular PM network formation by two main molecular mechanisms: [i] reducing PI(4,5)P_2_ levels through PLC activation; and [ii] inducing cortical F-actin mesh (via cortactin) and actomyosin (via ROCK1) formation. Rac1-mediated cortactin recruitment is insufficient for tubule inhibition, and requires actomyosin formation (myosin activation). Rac1-induced actomyosin formation prevents PI(4,5)P_2_-dependent tubule establishment either by an actively actin-dependent tubule scission process (Fig. 8, point 5) or by generating a cortical actomyosin network that produces a local mechanical barrier or increases PM tension to impede membrane invagination (Fig. 8, point 4).

For the first time, we identify ROCK1 as a novel downstream effector of Rac1 acting in a RhoA-independent manner to regulate membrane dynamics during a tubular CIE. Both Rac1 and RhoA GTPases stimulate actomyosin formation, but at different times and locations within the cell, and it is possible that both proteins share or compete for ROCK1. These results suggest that Rac1 activation at the leading edge of migrating cells may be important to stabilize β1-integrin in the newly generated adhesions, preventing its internalization and turnover, and therefore facilitating cell movement as a result.

## Material and Methods

### Reagents and Antibodies

W13 hydrochloride, U73122 and latrunculin A were from Calbiochem (Merck Millipore). Y27632, blebbistatin, neomycin and dynasore were from Sigma-Aldrich. DiC8-PI(4,5)P_2_ was from Echelon Biosciences. Primary antibodies used were as follows: rabbit polyclonal anti-GFP and mouse anti-actin (Abcam), mouse monoclonal anti-cortactin (Upstate), rabbit polyclonal anti-PACSIN2 (Abgent), rat monoclonal anti-β1-Integrin (AIIB2) (Damsky, C.H., Developmental Studies Hybridoma Bank), mouse monoclonal anti-PI(4,5)P_2_ (Echelon Biosciences), mouse monoclonal anti-early endosomal antigen1 (EEA1) (BD Transduction Laboratories), rabbit polyclonal anti-Myosin Heavy Chain IIA (Biolegend Inc.), rabbit polyclonal anti-Clathrin heavy chain (CHC) antibody (#PA5-25804) and transferrin-Alexa546 (#T23364) from ThermoFisher Scientific, and mouse monoclonal anti-CHC antibody clone ×22 (#MA1-065, Affinity BioReagents). Phalloidin conjugated with TRITC or Alexa-350, secondary Alexa-labeled antibodies and ProtA-HRP were from Molecular Probes (Invitrogen-Life Technologies). SiR actin (SC006) was from Spirochrome. Secondary HRP-labeled antibodies, SDS–polyacrylamide gel electrophoresis (PAGE) and molecular weight markers were from Bio-Rad. Glutathione-Sepharose beads were purchased from GE Healthcare. Human ROCK1 (4390824) siRNA was from Ambion, Human dynamin (s12097) siRNA was from Santa Cruz Biotechnologies, Human cortactin (CTTN, GS2017) and Human Clathrin heavy chain (CLTC, GS1213) Flexitube Gene Solution siRNAs were from Qiagen.

### Cell culture

African green monkey kidney fibroblast COS1 or Vero cells were grown in Dulbecco’s modified Eagle’s medium (DMEM) supplemented with 5% (v/v) or 10% fetal calf serum (FCS) respectively, pyruvic acid, antibiotics and glutamine. DMEM and FCS were purchased from Biological Industries.

### Plasmids and transfection

Plasmid encoding the constitutively active Rac1 mutant (Rac1^G12V^) was kindly provided by Dr Michiyuki Matsuda (University of Kyoto)^99^ and subcloned into living color vectors (Clontech). Rac1^F37A^ point mutation was introduced into pEGFPC1-Rac1^G12V^ by polymerase chain reaction (PCR) with the following primers: 5′-CAGATTCACCGGTTTTCCATCTACCATAACATTGGCAGAATAATTGTCAGCGACAGTAGGG-3′ and 5′-GGACGAGCTGTACAAGTCCCTCATGCAGGCCATCAAGTG-3′, using *BsrGI* and *BstXI* restriction sites; Rac1^W56A^ point mutation was introduced into pEGFPC1-Rac1^G12V^ by polymerase chain reaction (PCR) with the following primers: 5′-CAATTATTCTGCCAATGTTATGGTAGATGGAAAGCCAGTGAATCTGGGCTTAGCGGATACAGCTGG-3′ and 5′-CAGTCACGATGAATTCTTACAACAGCAGGC-3′, using *BstXI* and *EcoRI* restriction sites. Both mutants were subcloned into mCherry vector (Clontech). GFP- and Cherry-mem are fusion proteins that contain the N-terminal amino acids of GAP-43 and a GFP and mCherry fluorescent protein, respectively. The GAP-43 fragment contains a signal for post-translational palmitoylation of cysteines 3 and 4 that targets fusion protein to cellular membranes. Plasmid encoding dominant negative dynamin (dynamin-K44A) was obtained from ATCC (MBA-93). L10-GFP-Inp54p and L10-GFP-Inp54p^D281A^ were cloned from plasmids provided by Dr Tobias Meyer through Addgene (#20155 and #20156 respectively)^100^. Firstly, a peptide containing the N-terminal 10 residues of Lck (L10) was fused to the N-terminal GFP encoding sequence (L10-EGFP-C1); then, vectors from addgene were digested with *EcoRI* and *BamHI* and inserted into L10-EGFP-C1 vector. Cortactin-WT and Cortactin-ΔPHSH3 were kindly provided by Dr. Thomas Parsons. Plasmid encoding GST-ROCK1^725–1024^ was generated by PCR using the primers 5′-GACCGGTGGATCCCGGGCTGTATTAGCTTTCTTTCTATC-3′ and 5′-CACATGGTCCTGCTGGAGTTCGTG-3′ with pECFP-ROCK1, kindly provided by Dr Gareth Jones^101^, as a template. The resulting PCR product was introduced into CFP-N1 vector using *XhoI* and *XmaI* restriction sites, and then it was subcloned into pGEX-4T-2 for the expression in bacteria using *BamHI* and *XmaI* restriction sites. GFP-RhoA constitutively active (G14V) and dominant negative (T19N) were kindly provided by Michael Way (Cancer Research UK, London, UK). COS1 and Vero cells were transfected with DNA using Effectene (QIAGEN) or GenJet (Signagen), and transfected with combined DNA and siRNA transfection using RNAiMAX (Invitrogen-Life Technologies). Cells were used for experiments 24 h after DNA transient expression or 48–72 h after siRNA transfection.

### Immunofluorescence staining

Cells grown on coverslips were fixed with freshly prepared 4% paraformaldehyde (PFA) in cytoskeleton buffer (CB; 10 mM MES pH6.1, 138 mM KCl, 3 mM MgCl2, 2 mM EGTA) at 37 °C for 15 min and permeabilized with 0.5% Triton X-100 in CB at room temperature for 3 min. After 5-min incubation with blocking solution (TBST, 2% BSA), coverslips were incubated with the primary antibody diluted in blocking solution for 50 min at room temperature, washed intensively and then incubated with the adequate secondary antibodies labeled with Alexa488, Alexa555 or Alexa647. After staining, the coverslips were mounted in Mowiol (Calbiochem, Merck). The images were acquired using a Leica TCS SP5 laser scanning confocal microscope (Leica Microsystems Heidelberg GmbH) equipped with DMI6000 inverted microscope, Argon (458/476/488/514), diode pumped solid state (561 nm) and HeNe (633) lasers. GFP, TRITC or Alexa-555 and Alexa-647 images were acquired sequentially using 488, 561 and 633 laser lines, acoustic optical beam splitter (AOBS) as beam splitter, and emission detection ranges 500–555, 571–625 and 640–700 nm, respectively. STED confocal images were acquired using a Leica TCS SP8. Final analysis of all images was performed using IMAGEJ software.

### β1-integrin internalization analysis

COS1 cells grown on coverslips were tempered to 4 °C to defuse endocytosis and then were incubated with anti-β1-integrin antibody and transferrin-TRITC for 30 min at 4 °C. After washing the unbound antibody and transferrin excess with PBS, cells were incubated at 37 °C for 5, 10 and 20 min under the corresponding treatment. Cells were washed twice with PBS at 4 °C and then were subjected to a surface acid wash (0.5% glacial acetic acid, 0.5 M NaCl, pH 3.0) at 4 °C for 2 min. After fixation with freshly prepared 4% PFA at 37 °C for 15 min, immunostaining of the antigen-antibody complexes was performed as described above. Images were acquired along the Z-axis, in order to cover the whole cell, using a Leica TCS SP5 laser scanning confocal microscope (Leica Microsystems Heidelberg GmbH) equipped with DMI6000 inverted microscope. To determine the amount of internalized β1-integrin and transferrin, fluorescence intensity was normalized per cell area.

### Pull-down assay

Cleared TGH (1% Triton X-100, 10% glycerol, 50 mM Hepes with proteases inhibitors and 50 mM NaCl) lysates of COS1 cells, transiently expressing GFP-tagged Rac1 constructs, were split and incubated for 2 h at 4 °C with GST-ROCK1-725-1024 bound to gluthation-Sepharose beads. The unbound fraction was collected by centrifugation, and the remaining bound fraction was washed twice in lysis buffer supplemented with 150 mM NaCl and then once without NaCl. The total of the bound fraction was resolved by electrophoresis, and the proteins of interest were detected by western blotting.

## Electronic supplementary material

Supplemental Information
