# Supplementary material for: ROCK1 is a novel Rac1 effector to regulate tubular endocytic membrane formation during clathrin-independent endocytosis
Source: Sci Rep. 2017 Jul 31;7:6866. doi: 10.1038/s41598-017-07130-x (PMC5537229; doi:10.1038/s41598-017-07130-x)

SUPPLEMENTARY INFORMATION

ROCK1 is a novel Rac1 effector to regulate tubular endocytic membrane formation during clathrin-independent endocytosis

David Soriano-Castell[1], Albert Chavero[1], Carles Rentero[1], Marta Bosch[1], Maite Vidal-Quadras[1], Albert Pol[1,2], Carlos Enrich[1] and Francesc Tebar[1*].

[1] Departament de Biomedicina, Unitat de Biologia Cel·lular, Centre de Recerca Biomèdica CELLEX, Institut d'Investigacions Biomèdiques August Pi i Sunyer (IDIBAPS), Facultat de Medicina, Universitat de Barcelona, Casanova 143, 08036-Barcelona, Spain.

[2] Institució Catalana de Recerca i Estudis Avançats (ICREA), 08010-Barcelona, Spain.

Supplementary Figure Legends

Supplementary Figure S1. Rac1^{G12V} inhibits W13-induced PACSIN-positive tubules. COS1 cells (1 hour starved) expressing GFP-Rac1^{G12V} grown in coverslips and untreated (NT) or treated with W13 (20 min, 4.5 µg/ml), PACSIN2 was detected by immunofluorescence with an antibody and the corresponding Alexa-555 anti-rabbit secondary antibody (bars, 10 µm).

Supplementary Figure S2. Active Rac1 reduces PI(4,5)P$_2$ levels in W13-treated cells. Endogenous PI(4,5)P$_2$ was detected by immunofluorescence in COS1 cells expressing Cherry-Rac1^{G12V} and incubated with or without W13, using an antibody and the corresponding Alexa-488 anti-mouse secondary antibody (bars, 10 µm).

Supplementary Figure S3. ROCK1 activity does not participate in cortactin translocation to the PM induced by active Rac1. Cortactin was detected by immunofluorescence in 1-hour starved Vero cells expressing GFP-Rac1^{G12V} or GFP-Rac1$^{G12V-W56A}$ after treatment with the ROCK1 inhibitor Y27632 (20 min, 25 µM). Magnification insets show cortactin localized at the plasma membrane in both cases (bars, 5 µm).

Supplementary Figure S4. W13-induced tubule network do not colocalize with F-actin and follow the pattern of microtubules, which are necessary for increased integrin internalization. a) COS1 cells expressing Venus-Rac1wt grown on coverslips were incubated with W13 (20 min, 4,5 µg/ml). After fixation (PFA 4%, 15 min at 37°C), β-tubulin was detected by immunofluorescence with a mouse primary antibody and the corresponding anti-mouse Alexa-594 labeled secondary antibody. F-actin was detected using SiR-actin (SC006). Several projection images acquired with a confocal microscope (Leica TCS SP5) are shown (bars, 10 µm). **b, c)** COS1 cells grown on coverslips expressing the membrane marker GFP-mem were incubated with an anti-β1-integrin rat antibody and transferrin-TRITC for 30 minutes at 4°C to avoid endocytosis, followed by incubation for 10 minutes at 37°C to allow internalization in the presence of W13 (20 min, 4.5 µg/ml) and/or nocodazole (30 µM). After fixation, β1-integrin was detected with an Alexa-647 labeled anti-rat antibody, and images were acquired with a confocal microscope (Leica TCS SP5). Quantification of internalized β1-integrin (b) and transferrin (c), as explained in the *Materials and Methods,* in the indicated conditions (W13t, cells presenting tubules; W13nt, cells without tubules) is shown. Mean values ± standard error of the mean (SEM) from three independent experiments are shown. Statistical significance between different conditions and the corresponding control was determined by Student's *t*-test, $*p < 0.05$.

Figure Supplemental 1

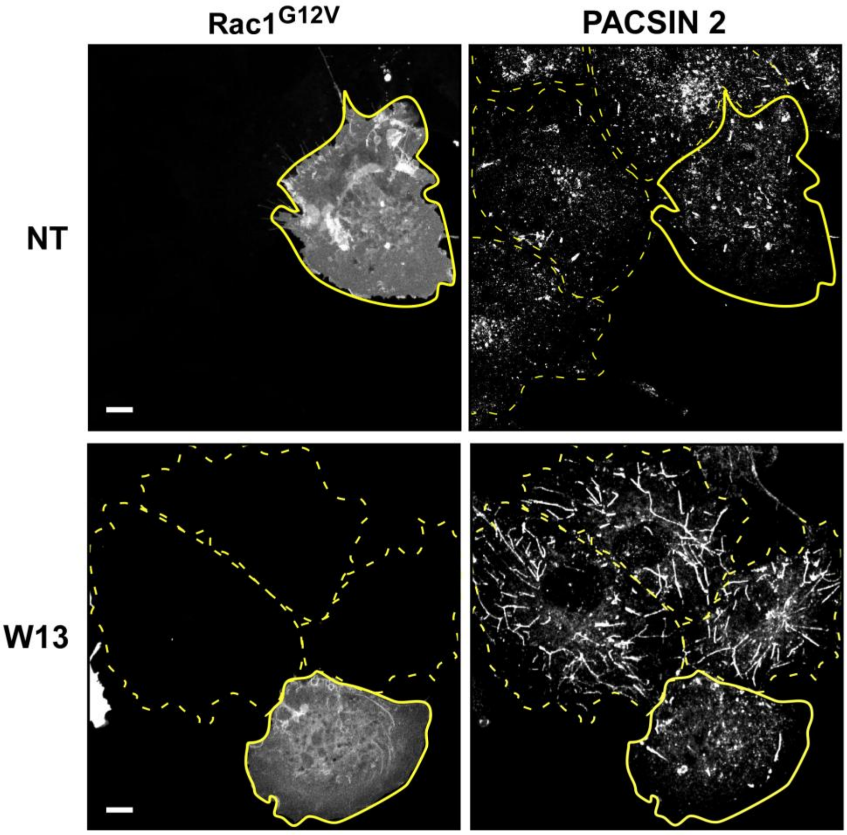

Figure Supplemental 2

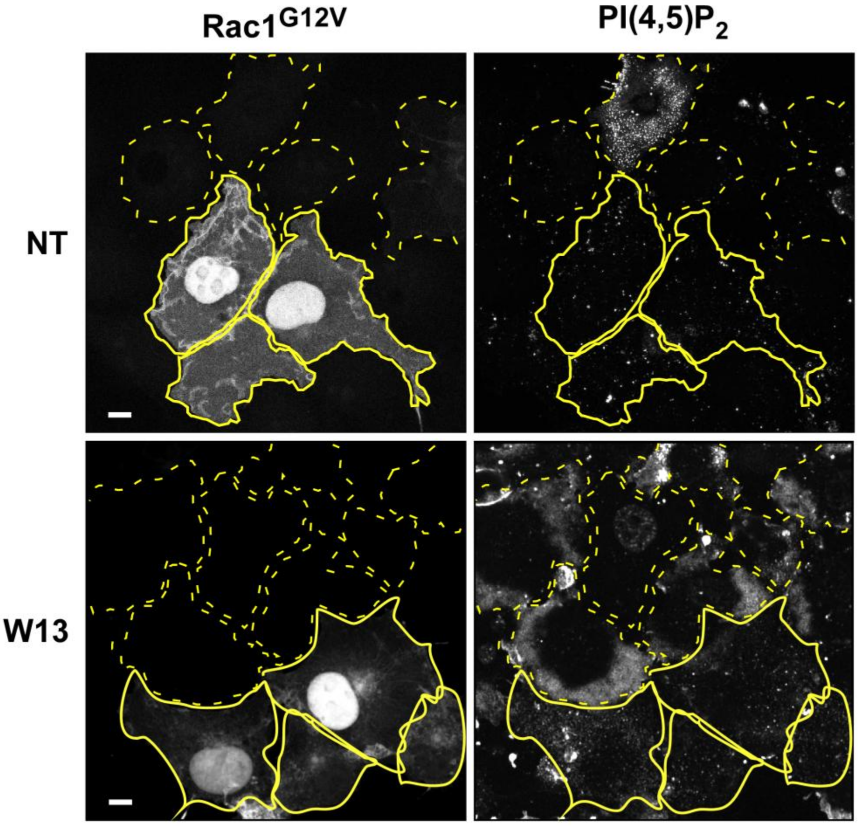

Figure Supplemental 3

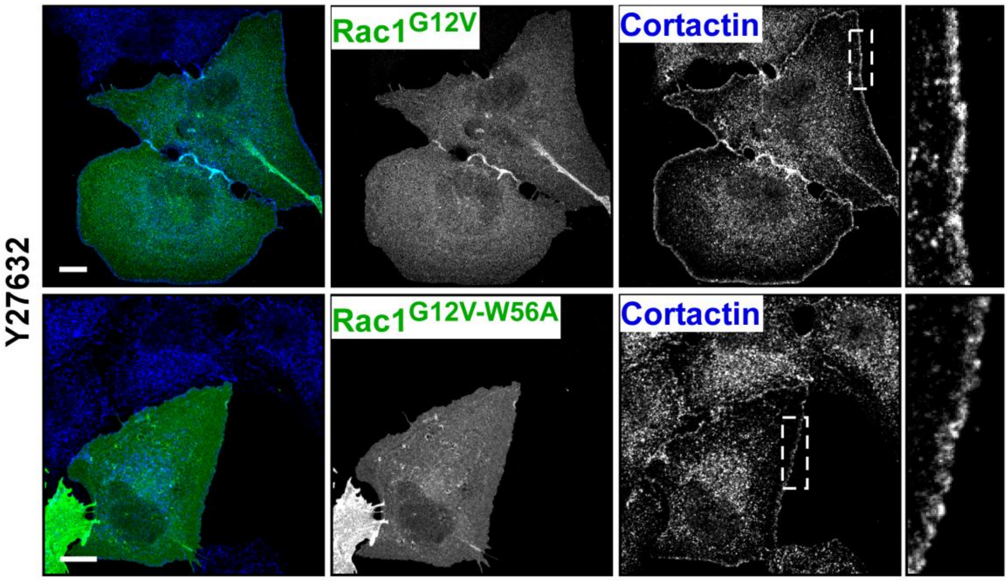

Figure Supplemental 4

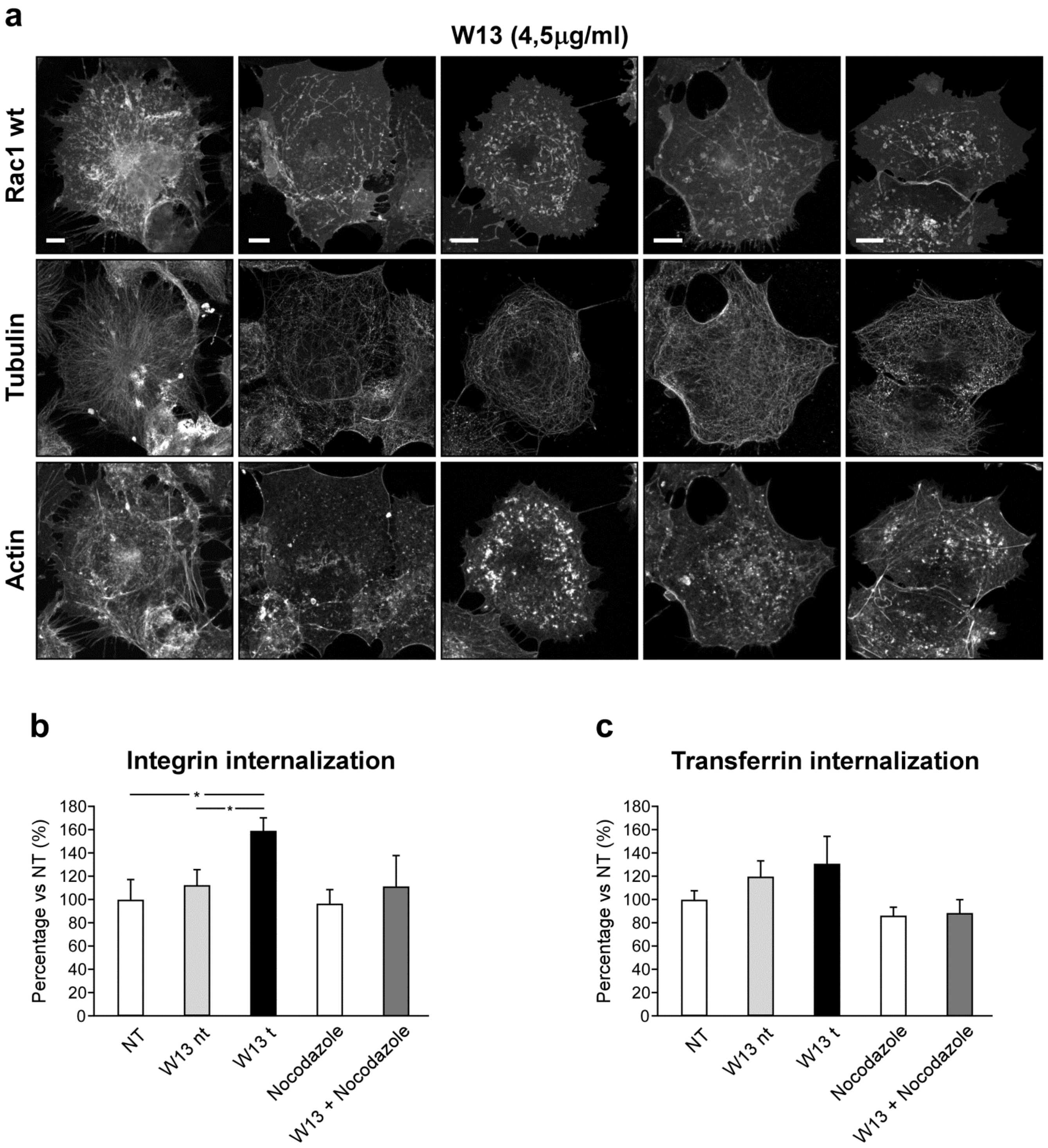

Supplement: Supplementary file 1 — Supplemental Information [file 41598_2017_7130_MOESM1_ESM.pdf]
